# Ruminal and postruminal absorption of copper, manganese, and zinc of nonlactating and nonpregnant Holstein cows fed sulfate trace minerals above recommendations

**DOI:** 10.3168/jdsc.2025-0780

**Published:** 2025-06-12

**Authors:** M. Duplessis, D.R. Ouellet, I. Royer, R.M. Petri

**Affiliations:** 1 Agriculture and Agri-Food Canada, Sherbrooke Research and Development Centre, Sherbrooke, Québec, J1M 0C8, Canada; 2 Agriculture and Agri-Food Canada, Québec Research and Development Centre, Québec, Québec, G1V 2J3, Canada

## Abstract

The objective of this study was to evaluate ruminal and postruminal apparent absorption of Cu, Mn, and Zn with rations containing 2 different concentrations of sulfate Co, Mn, and Zn supplements in nonlactating, nonpregnant Holstein cows. Four multiparous cows with ruminal and duodenal cannulas were used in a double 2 × 2 crossover design. Cows were assigned to the following treatments: (1) mineral supplement providing concentrations of Co, Mn, and Zn at 0.10, 16.7, and 60.8 mg/kg DM, respectively (CON) and (2) mineral supplement providing high concentrations of Co, Mn, and Zn at 0.63, 96.3, and 139.7 mg/kg DM, respectively (HTM). After 20 d of diet adaptation, ruminal, duodenal, and fecal samples were collected during 5 consecutive days to represent every 2 h of the day. Diets and orts were collected daily. All samples were analyzed for Cu, Mn, and Zn concentrations. Two markers (chromic oxide and indigestible NDF) were used to evaluate duodenal digesta outflow and feces output. Dry matter intake was similar between treatments (averaged 17.7 kg/d) and DM digestibility tended to be lower by 3% for HTM than CON cows. Regardless of the marker used, the interpretation of results for ruminal, postruminal, and total apparent absorption remained similar. Ruminal and postruminal absorption of Cu, Mn, and Zn did not differ between treatments. Total apparent absorption of Cu was lower for HTM than CON cows, but no treatment effect was noted for Mn and Zn. Zinc was absorbed both before and after the duodenum cannula. This article gives a useful insight on the potential site of absorption of sulfate Cu, Mn, and Zn in nonlactating and nonpregnant Holstein cows under a nonrestrictive supply of trace minerals.

## Highlights

• Dry matter digestibility tended to be smaller with greater dietary Mn and Zn.
• Regardless of the digestive marker, results remained similar.
• Apparent absorption of Cu was smaller with greater dietary Mn and Zn concentrations.
• This provides insights into the potential sites of absorption of Cu, Mn, and Zn in dairy cattle.

## Graphical Abstract

**Figure:**
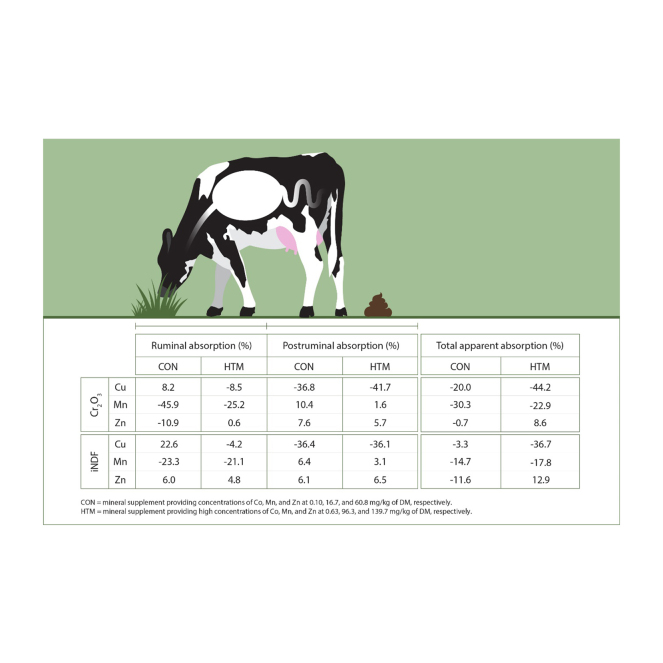
**Summary:** Our understanding of trace mineral absorption throughout the ruminant gut is limited. Four nonlactating and nonpregnant multiparous Holstein cows with ruminal and duodenal cannulas were used in a crossover design to evaluate ruminal, postruminal, and total Cu, Mn, and Zn apparent absorption at 2 dietary Co, Mn, and Zn supplies based on 2 digestive markers (chromium oxide [Cr_2_O_3_] and indigestible neutral detergent fiber [iNDF]). Copper, Mn, and Zn were respectively fed 41%, 31%, and 283% (control) and 48%, 418%, and 676% (high) above cow requirements. Regardless of the digestive marker used, the interpretation of results remained similar. Apparent absorption of Cu was smaller with greater dietary Mn and Zn, although no effect was noted on ruminal and postruminal Cu absorptions. Zinc was absorbed before and after the duodenum cannula. Apparent absorption of Zn averaged 12%. This article gives insight into the potential absorption sites of Cu, Mn, and Zn at maintenance.

Trace minerals (**TM**) play vital roles in mammalian metabolism; thus, dietary supply is important. However, cow requirements are uncertain. In NASEM (2021), dietary Cu, Mn, and Zn recommendations are based on a factorial approach, which is the sum of nutrients required for maintenance, growth, milk production, and gestation, if any. This approach is based on absorbed minerals that should then be divided by an absorption coefficient to obtain the dietary recommendations (Weiss and Hansen, 2024). Research to determine absorption coefficients of TM based on different dietary factors is scarce. In addition, our understanding of the TM absorption throughout the gut of ruminants is limited. Most of the time, data on absorption coefficients come from old studies or studies on rodents, which do not necessarily represent dairy cattle. As a result, it is a common practice to feed excess TM to dairy cows as an insurance to fulfill their requirements (Duplessis et al., 2023).

Most TM are assumed to be absorbed in the small intestine (Goff, 2018), and it has been suggested that Zn was also absorbed by the rumen wall (NRC, 2005). Little information is available on Mn absorption in dairy cows. It has also been suggested that Cu and Zn absorptions are dependent on dietary Cu and Zn supply because absorption is optimized as their dietary intake is closer to the animal requirements (NRC, 2005). Nevertheless, absorption of these nutrients in ruminants is recognized to be low, largely due to interactions that occur in the rumen (NRC, 2005). Determining TM absorption with accuracy and precision is difficult in ruminants because of the small quantities required by the animals, the low absorption, and the technical challenge of the laboratory analysis. Small errors during sampling or in the laboratory can lead to a tremendous error in the estimation of apparent absorption.

This study was conducted to evaluate ruminal and postruminal apparent absorption of Cu, Mn, and Zn with rations containing 2 different concentrations of sulfate Co, Mn, and Zn supplements in nonlactating and nonpregnant Holstein cows. Copper was also studied, although there was no change between treatments in Cu concentration to evaluate possible interactions with other TM. Results related to Co were not presented due to an issue of sensitivity of the analysis (NASEM, 2021).

All procedures on animals were previously approved by the Institutional Animal Care Committee of the Sherbrooke Research and Development Centre (Sherbrooke, QC, Canada; internal number #575) under the guidelines of the Canadian Council on Animal Care (2009).

Four multiparous nonlactating and nonpregnant Holstein cows (779 ± 43 kg) were used in a double 2 × 2 crossover design. The trial was conducted at the research facilities barn of the Sherbrooke Research and Development Centre on cows already fitted with a rumen cannula (10 cm; Bar Diamond Inc., Parma, ID) and a duodenal closed T-type cannula placed approximately 10 cm distal to the pylorus. Cows were housed in tiestalls under 17 h of light per day. Rations used in the present experiment were purposely formulated for the trial of Marchand et al. (2024) aiming to evaluate the effects of short-term feeding of sulfate TM above the recommendations on lactating dairy cows. Rations were conveniently used in the current assessment on 4 supplemental nonlactating and nonpregnant cows already equipped with a duodenal cannula to get an insight into postruminal absorption of TM. Cows received the same diet except the concentrations of the sulfate TM supplement differed as follows: (1) TM supplement aimed to provide concentrations of Co, Mn, and Zn based on the NRC (2001) recommendations of a 650-kg cow producing 40 kg of milk/d with a DMI of 20 kg/d (**CON**; expected: 0.11, 17, and 63 mg/kg of DM, respectively; actual: 0.10, 16.7, and 60.8 mg/kg of DM, respectively) and (2) TM supplement aimed to provide concentrations of Co, Mn, and Zn above the NRC (2001) recommendations (**HTM**; expected: 0.95, 114, and 123 mg/kg of DM, respectively; actual: 0.63, 96.3, and 139.7 mg/kg of DM, respectively) as per the 90th percentile of supplemental TM concentration of the study of Duplessis et al. (2021) in Canadian dairy herds. Other nutrients were formulated according to the Cornell Net Carbohydrate and Protein System software (CNCPS version 6.5/6.55; Cornell University, Ithaca, NY) from NDS Professional software (RUM&N Sas, Reggio Emilia, Italy). Diets were composed of about 32.9% of corn silage, 30.0% of grass silage, and 19.1% of cracked corn on a DM basis (Marchand et al., 2024). Diets were served once daily at 0900 h and 40 kg as fed (DM of TMR at about 45%) was offered due to the low energy requirements of nonlactating and nonpregnant cows. Cows had free access to water. Each period lasted 25 d and sampling collection started after 20 d of adaptation to the diet. Between the 2 periods, there was a 20-d period of wash-out in which cows received dry hay only. The barn staff were not blinded to the treatments.

Four days before sampling collection, at d 17, a gelatin bolus containing 8 g of chromic oxide (Cr_2_O_3_) was administered directly through the ruminal cannula twice daily at 12 h apart, for a daily dose of 16 g of Cr_2_O_3_ and continued throughout the sampling period until d 25. At d 21, sampling collection of ruminal (1 L), duodenal (0.5 L), and fecal (500 g) samples started and lasted until d 25 according to the following schedule to get a fair representation of the daily rhythm of the animal: at 0800 and 1600 h on d 21; at 0000, 1000, and 1800 h on d 22; at 0200, 1200, and 2000 h on d 23; at 0400, 1400, and 2200 h on d 24; and at 0600 h on d 25. Whole-rumen samples were collected from 5 rumen locations (anterior dorsal, anterior ventral, medium ventral, posterior dorsal, and posterior ventral) and pooled. Feces were obtained either naturally or by grab sampling. From d 21 to 25, orts were weighed daily. All ingredients of the ration and orts were sampled for nutrient composition analyses as previously reported (Marchand et al., 2024). All samples were stored at −20°C until processing.

Samples were thawed at room temperature, dried in an air-forced oven at 55°C for 72 h, and ground at 1 mm. All collected samples were individually analyzed for their Cr, Cu, Mn, and Zn concentrations. For Cu, Mn, and Zn analyses, 1.0 g of diet, ort, ruminal, and duodenal samples and 0.5 g of feces, dried and ground, were weighed in duplicate in glass tubes and placed in a muffle furnace at 550°C for 18 h for ash determination (method 942.05; AOAC International, 2005). Samples were then solubilized with 4 mL of HCl 6 *N*, heated at 70°C for 2 h, and filtered with a filter paper (Grade 54; Cytiva Whatman, Marlborough, MA) in 50-mL tubes. They were rinsed with HCl 3 *N* and filtered again with the filter paper until solutions of 25 mL were obtained. The paper filter was rinsed with ultra-pure water, removed, and ultra-pure water was added until solutions of 50 mL were obtained. Each duplicate sample was analyzed by atomic absorption spectrometer with an air-acetylene flame (PinAAcle 900F, PerkinElmer, Woodbridge, ON, Canada) and read 4 times. Chromium analysis was performed as previously described by Siddons et al. (1985) with slight modifications. Briefly, 0.5 g of duodenal and fecal samples were weighed in duplicate in glass tubes and incinerated at 550°C for 18 h. Three milliliters of digestion acid (30 mL of 7.58% wt/vol MnSO_4_ into 1 L of 85% H_3_PO_4_) and 4 mL of 4.5% (wt/vol) BrKO_3_ solution were added to the glass tubes, which were covered by a marble and digested on a heated plate for approximately 70 min until effervescence ceased. After a waiting time of 30 s, 6 mL of CaCl_2_ solution was added (14.7 g of CaCl_2_ in 1 L of ultra-pure water). The content of each glass tubes was transferred into 50-mL tubes and ultra-pure water was added to get a final volume of 50 mL. Each duplicate sample was analyzed by an atomic absorption spectrometer with an air-acetylene flame and read 4 times. Intraassay CV of 1.7%, 1.5%, 1.9%, and 1.8% were obtained for Cr, Cu, Mn, and Zn concentrations, respectively. For indigestible NDF (**iNDF**) analysis, refusal, duonenal, and fecal samples were composited by cow per period, and TMR samples were composited by treatment per period after sample drying and grounding. For TMR, refusals, and feces, 0.5 g was weighed and, for duodenal samples, 1.0 g was weighed in fiber filter bags (F58, Ankom Technology, Macedon, NY) that were incubated in the ventral part of the rumen for 288 h in 2 lactating Holstein cows equipped with ruminal cannulas. For each cow, each composite sample was weighed in 3 individual filter bags. Cows were fed once daily at 0900 h of a ration containing 32.5% of corn silage, 32.4% of grass-legume silage, 18.0% of cracked corn, 6.3% of energy supplement (distiller grain, corn gluten meal, canola meal, and micronized soybean), 5.3% of soybean meal, 2.5% of minerals and vitamins, 1.6% of grass hay, and 1.4% of rumen inert fat supplement. After 144 h of incubation, ruminal cannulas were opened to make sure that filter bags were still in the ventral part of the rumen and submerged by ruminal liquid. After 288 h of incubation, filter bags were cold washed as described by Morris et al. (2018). Then, each filter bag was dried at 55°C for 48 h, and NDF concentration was analyzed as described by Van Soest et al. (1991) using sodium sulfite and heat stable α-amylase to determine iNDF. Data from the 2 cows were then averaged. Chromium and iNDF were respectively used as inert external and internal markers to estimate duodenal digesta outflow and fecal output (Huhtanen et al., 1994; Titgemeyer, 1997).

Data were averaged by cow by period using Proc SQL of SAS (SAS Institute, 2012). For both markers, duodenal digesta outflow and fecal output were calculated as follows: quantity of marker ingested or administered (Cr or iNDF; g/d)/concentrations of the marker in duodenal or fecal samples (Cr or iNDF; g/g of DM). Then, estimated daily duodenal digesta outflow and fecal output of Cu, Mn, and Zn were obtained by multiplying duodenal digesta outflow or fecal output by their respective duodenal or fecal concentrations. Ruminal Cu, Mn, and Zn absorption was estimated as follows: [(daily intake − daily duodenal digesta outflow)/daily intake]. Postruminal Cu, Mn, and Zn absorption was estimated by [(daily duodenal digesta outflow − daily fecal output)/daily duodenal digesta outflow]. Total Cu, Mn, and Zn apparent absorption was obtained by dividing intestinal disappearance by their daily intake. Copper, Mn, and Zn intestinal disappearances were obtained by subtracting their daily intake from their fecal output. To validate our methodology of utilizing Cr_2_O_3_ as an external marker for estimating fecal output and iNDF as an internal marker for calculating DM digestibility, actual DMI were compared with the estimates derived from the approach used by Velásquez et al. (2018), who successfully validated this technique for estimating DMI. Actual DMI was calculated by intake minus orts on a DM basis.

For each marker, data were analyzed separately according to a crossover design in Proc MIXED of the SAS software. Fixed effects were dietary treatments and periods, and cow was considered a random effect. To compare results pertaining to DMI, duodenal digesta outflow, and feces output obtained with Cr_2_O_3_ and iNDF as a marker, Proc MIXED of SAS was used with cow, dietary treatments, periods, markers (either Cr_2_O_3_ or iNDF), and treatments × markers interaction as fixed effects. Markers were considered as repeated measurement with cow nested with period as a subject. Several covariance structures (compound symmetry, heterogeneous compound symmetry, first-order autoregressive, and heterogeneous first-order autoregressive) were tested and the one leading to the lowest Bayesian information criterion was retained. Normality and homoscedasticity were visually assessed with the residuals. The 2-sided TTEST procedure was performed in SAS to evaluate if absorptions were significantly different from 0. Significant effects were declared at *P* ≤ 0.05 and tendencies at *P* between 0.05 and 0.10. Results using the 2 markers were presented separately because no literature exists on the use of markers to evaluate ruminal and postruminal absorption of the selected TM. Moreover, it allows us to make a comparison between the 2 markers.

Actual DMI and refusals averaged 17.7 (SEM: 0.1) and 1.0 (SEM: 0.7) kg/d and did not differ between treatments (*P* > 0.18). Estimated DMI using both external and internal markers was 18.1 (SEM: 0.7) kg/d and was not different from the actual DMI (marker effect, *P* = 0.52, Table 1). Dry matter digestibility averaged 62% and was similar to data reported by Velásquez et al. (2018) with the use of markers. There was a tendency for a lower DM digestibility for HTM cows (*P* = 0.10). Although Marchand et al. (2024) did not report any deleterious effects of feeding excess TM on lactating cows, feeding the same concentrations of TM to nonlactating and nonpregnant cows, whose nutrient requirements are only based on maintenance, can have a different impact on digestibility. Based on maintenance requirements of nonlactating and nonpregnant CON cows (NASEM, 2021), daily intakes of 251 mg of Cu, 482 mg of Mn, and 440 mg of Zn were required. Hence, CON cows were fed 41%, 31%, and 283% and HTM were fed 48%, 418%, and 676% above Cu, Mn, and Zn requirements, respectively.

**Table 1:** Dry matter intake and digestibility, and dietary, ruminal, duodenal, and fecal concentrations of Cu, Mn, and Zn of nonlactating and nonpregnant Holstein dairy cows

Item	Treatment		SEM	*P*-value
	CON^1^	HTM^2^		
DMI (kg/d)				
Actual	17.6	17.8	0.1	0.18
Estimated^3^	17.7	18.4	0.7	0.52
DM digestibility^4^ (%)	63.0	61.4	0.5	0.10
Cu (mg/kg DM)				
Diet	22.3	21.4	—	—
Rumen	24.9	26.8	1.3	0.19
Duodenum	25.6	31.4	1.7	0.14
Feces	63.4	76.1	3.0	0.03
Mn (mg/kg DM)				
Diet	39.8	142.8	—	—
Rumen	33.6	90.3	2.3	0.002
Duodenum	72.2	242.8	10.6	0.003
Feces	125.4	439.1	21.2	0.001
Zn (mg/kg DM)				
Diet	98.9	194.2	—	—
Rumen	103.7	155.1	3.6	0.01
Duodenum	147.9	260.5	17.3	0.04
Feces	257.2	445.9	9.3	0.005

1 CON = control group. The sulfate TM supplement aimed to provide concentrations of Co, Mn, and Zn as recommended by the NRC (2001) based on a 650-kg cow producing 40 kg of milk/d with a DMI of 20 kg/d (0.11, 17, and 63 mg/kg DM, respectively) based on the study of Marchand et al. (2024). The sulfate TM supplement actually provided 0.10 mg of Co/kg of DM, 13.2 mg of Cu/kg of DM, 42.2 mg of Fe/kg of DM, 16.7 mg of Mn/kg of DM, and 60.8 mg of Zn/kg of DM.

2 HTM = high trace mineral supplement. The sulfate TM supplement aimed to provide concentrations of Co, Mn, and Zn above the NRC (2001) recommendation (0.95, 114, and 123 mg/kg DM, respectively) based on the study of Duplessis et al. (2021) and Marchand et al. (2024). The sulfate TM supplement actually provided 0.63 mg of Co/kg of DM, 12.7 mg of Cu/kg of DM, 43.6 mg of Fe/kg of DM, 96.3 mg of Mn/kg of DM, and 139.7 mg of Zn/kg of DM.

3 Estimated as per Velásquez et al. (2018) using Cr_2_O_3_ as an external marker and iNDF as an internal marker.

4 Estimated as per Velásquez et al. (2018) using iNDF as an internal marker.

Ruminal, duodenal, and fecal concentrations of Mn and Zn were significantly greater for HTM than CON cows (*P* ≤ 0.04; Table 1). Ruminal and duodenal Cu concentrations were not different between treatments, whereas HTM cows had greater fecal Cu concentration than CON cows (*P* = 0.03).

Duodenal digesta outflow estimation did not differ between the 2 markers used (marker effect; *P* = 0.12). However, fecal output estimation was lower when iNDF was used as a marker (marker effect; *P* = 0.04; 6.2 [SEM: 0.2] kg DM/d for iNDF and 6.8 [SEM: 0.4] kg DM/d for Cr_2_O_3_). Nevertheless, regardless of the marker used, the interpretation of results for ruminal, postruminal, and total apparent absorption remained similar. The largest difference in treatment effect was observed for total Zn apparent absorption between the 2 marker methods (*P*-values of 0.11 and 0.76 for Cr_2_O_3_ and iNDF, respectively), but none of the *P*-values reached significance. Thus, the following results will be presented regardless of marker unless indicated otherwise.

Daily intake, duodenal digesta outflow, and fecal output of Mn and Zn differed by treatments (*P* ≤ 0.04; Table 2). Ruminal and postruminal absorption of Cu, Mn, and Zn did not differ between treatments (*P* ≥ 0.10). Total apparent absorption of Cu was lower for HTM than CON cows (*P* ≤ 0.04), but no treatment effect was noted for Mn and Zn (*P* ≥ 0.11). To avoid a surplus of free ionized Zn when excess Zn is supplied, metallothionein binds Zn (Goff, 2018). However, this process is not selective and also binds Cu, hence decreasing Cu absorption from the enterocyte. Moreover, a part of Cu, Mn, and Zn absorption can be accomplished by a common epithelial receptor, causing competition (Goff, 2018). It is stated in the NASEM (2021) that Zn antagonism for Cu absorption is not of practical importance because dietary Zn concentration should be 10 to 20 times the requirement before clinical evidence occurs. This is based on a study of Miller et al. (1989) that found plasma Cu concentration was reduced in cows fed 2,000 mg of Zn/kg of DM compared with 0 and 1,000 mg of Zn/kg of DM. It is noteworthy that plasma concentration is not a good indicator of dietary TM supply and is not sensitive enough to determine TM status (Weiss, 2017). Current results suggest that the dogma that excess Zn does not have a practical impact on Cu absorption should be reevaluated. However, it should be noted that the current total apparent Cu absorptions were very low and far from the estimated value of 5% used by the NASEM (2021). A small absorption coefficient was also observed for Mn, which appeared to be negative in the current assessment and 0.4% according to the NASEM (2021). Several hypotheses might be drawn to explain the current outcomes: (1) sampling errors leading to heterogeneous samples, (2) analytical errors due to the low concentrations of TM in samples (Weiss, 2017), (3) addition of sampling and analytical errors in the calculation of absorption, and (4) the use of digestive markers can also be a source of error in output estimation (Titgemeyer, 1997) instead of total feces collection. Daniel et al. (2023) also obtained slight negative total apparent Mn absorption in nonlactating and pregnant cows. Interestingly, ruminal absorption was greater for Cu than for Mn and postruminal absorption was greater for Mn than for Cu. From the results obtained, it can be hypothesized that Zn is both absorbed before and after the duodenum cannula. However, ruminal Zn absorptions were not significantly different from 0. Based on the iNDF marker, total apparent Zn absorption averaged 12.2%, which corroborates the previously reported result in nonlactating cows (diet provided 143% above requirements; Daniel et al., 2023) but lower than in lactating cows (diet provided 5% above requirements; Duplessis et al., 2025). To our knowledge, this is the first study evaluating both ruminal and postruminal absorption of TM. Thus, this article gives a useful insight on the potential site of absorption of Cu, Mn, and Zn in nonlactating and nonpregnant Holstein cows.

**Table 2:** Intake, duodenal digesta outflow, fecal output, ruminal and postruminal, and total apparent absorption of Cu, Mn, and Zn of nonlactating and nonpregnant Holstein dairy cows based on 2 different digestive markers

Item	Treatment		SEM	*P*-value
	CON^1^	HTM^2^		
With Cr_2_O_3_ as a marker				
Duodenal digesta outflow (kg/d DM)	12.6	12.9	0.4	0.54
Fecal output (kg/d DM)	6.6	7.1	0.3	0.31
Cu				
Intake (mg/d)	354	371	19	0.59
Duodenum (mg/d)	322	411	28	0.10
Feces (mg/d)	418	538	29	0.04
Ruminal absorption (%)	8.2	−8.5	8.8	0.26
Postruminal absorption (%)	−36.8	−41.7^3^	8.2	0.71
Total apparent absorption (%)	−20.0	−44.2^3^	7.5	0.04
Mn				
Intake (mg/d)	631	2,495	34	0.0005
Duodenum (mg/d)	907	3,136	183	0.002
Feces (mg/d)	813	3,080	180	0.0005
Ruminal absorption (%)	−45.9^3^	−25.2	11.8	0.25
Postruminal absorption (%)	10.4^3^	1.6	3.7	0.24
Total apparent absorption (%)	−30.3^3^	−22.9	9.5	0.44
Zn				
Intake (mg/d)	1,684	3,416	76	0.002
Duodenum (mg/d)	1,866	3,401	194	0.03
Feces (mg/d)	1,694	3,132	84	0.0002
Ruminal absorption (%)	−10.9	0.6	8.1	0.42
Postruminal absorption (%)	7.6	5.7	7.9	0.88
Total apparent absorption (%)	−0.7	8.6^3^	2.4	0.11
With iNDF as a marker				
Duodenal digesta outflow (kg/d DM)	10.8	12.4	0.8	0.22
Fecal output (kg/d DM)	5.8	6.7	0.4	0.26
Cu				
Intake (mg/d)	354	371	19	0.59
Duodenum (mg/d)	277	393	32	0.09
Feces (mg/d)	367	509	30	0.06
Ruminal absorption (%)	22.6^3^	−4.2	6.6	0.10
Postruminal absorption (%)	−36.4^3^	−36.1^3^	4.7	0.97
Total apparent absorption (%)	−3.3	−36.7^3^	4.0	0.03
Mn				
Intake (mg/d)	631	2,495	34	0.0005
Duodenum (mg/d)	782	3,030	179	0.005
Feces (mg/d)	731	2,953	164	0.0001
Ruminal absorption (%)	−23.3^3^	−21.1	8.2	0.79
Postruminal absorption (%)	6.4	3.1	3.7	0.51
Total apparent absorption (%)	−14.7^3^	−17.8	4.8	0.36
Zn				
Intake (mg/d)	1,684	3,416	76	0.002
Duodenum (mg/d)	1,600	3,244	249	0.04
Feces (mg/d)	1,504	2,983	113	0.007
Ruminal absorption (%)	6.0	4.8	9.1	0.94
Postruminal absorption (%)	6.1^3^	6.5	5.6	0.97
Total apparent absorption (%)	11.6	12.9^3^	4.4	0.76

1 CON = control group. The sulfate TM supplement aimed to provide concentrations of Co, Mn, and Zn as recommended by the NRC (2001) based on a 650-kg cow producing 40 kg of milk/d with a DMI of 20 kg/d (0.11, 17, and 63 mg/kg DM, respectively) based on the study of Marchand et al. (2024). The sulfate TM supplement actually provided 0.10 mg of Co/kg of DM, 13.2 mg of Cu/kg of DM, 42.2 mg of Fe/kg of DM, 16.7 mg of Mn/kg of DM, and 60.8 mg of Zn/kg of DM.

2 HTM = high trace mineral supplement. The sulfate TM supplement aimed to provide concentrations of Co, Mn, and Zn above the NRC (2001) recommendation (0.95, 114, and 123 mg/kg DM, respectively) based on the studies of Duplessis et al. (2021) and Marchand et al. (2024). The sulfate TM supplement actually provided 0.63 mg of Co/kg of DM, 12.7 mg of Cu/kg of DM, 43.6 mg of Fe/kg of DM, 96.3 mg of Mn/kg of DM, and 139.7 mg of Zn/kg of DM.

3 Significantly or tending to be (*P* < 0.10) different from 0 according to the TTEST procedure (SAS, version 9.4).

A limitation of the study is the low number of cows involved due to the fact that cows had to have a duodenal cannula. It is also noteworthy that cows in the current study only had maintenance requirements as they were neither lactating nor pregnant, were fed a lactation diet, and were eating more DM than with a regular dry cow diet. It can then be concluded that these cows received more TM than required. The short duration of each treatment period can be insufficient to fully evaluate the impact of different dietary concentrations of TM. Moreover, the current study did not evaluate if ruminal and postruminal TM absorptions implied actual absorption into the bloodstream. This should be kept in mind when interpreting the results.

## References

[bib1] AOAC International (2005).

[bib2] Canadian Council on Animal Care. 2009. Guide to the Care and Use of Experimental Animals. Vol. 1. 2nd ed. E. D. Olfert, B. M. Cross, and A. A. McWilliam, ed. Ottawa, ON, Canada.

[bib3] Daniel J.-B., Brugger D., van der Drift S., van der Merwe D., Kendall N.R., Windisch W.M., Doelman J., Martín-Tereso J. (2023). Zinc, copper, and manganese homeostasis and potential trace metal accumulation in dairy cows: Longitudinal study from late lactation to subsequent mid-lactation. J. Nutr..

[bib4] Duplessis M., Fadul-Pacheco L., Santschi D.E., Pellerin D. (2021). Toward precision feeding regarding minerals: What is the current practice in commercial dairy herds in Québec, Canada?. Animals (Basel).

[bib5] Duplessis M., Hassanat F., Côrtes C., Benchaar C. (2025). Apparent zinc absorption in Ayrshire and Holstein lactating cows. Anim. Open Space.

[bib6] Duplessis M., Wright T.C., Bejaei M. (2023). A survey of Canadian dairy nutritionists to assess current trace element formulation practices. J. Dairy Sci..

[bib7] Goff J.P. (2018). Invited review: Mineral absorption mechanisms, mineral interactions that affect acid-base and antioxidant status, and diet considerations to improve mineral status. J. Dairy Sci..

[bib8] Huhtanen P., Kaustell K., Jaakkola S. (1994). The use of internal markers to predict total digestibility and duodenal flow of nutrients in cattle given six different diets. Anim. Feed Sci. Technol..

[bib9] Marchand C., Royer I., Gervais R., Girard C.L., Benchaar C., Hassanat F., Zastepa A., Crevecoeur S., Duplessis M. (2024). Effects of feeding sulfate trace minerals above recommendations on nutrient digestibility, rumen fermentation, lactational performance, and trace mineral excretion in dairy cows. J. Dairy Sci..

[bib10] Miller W.J., Amos H.E., Gentry R.P., Blackmon D.M., Durrance R.M., Crowe C.T., Fielding A.S., Neathery M.W. (1989). Long-term feeding of high zinc sulfate diets to lactating and gestating dairy cows. J. Dairy Sci..

[bib11] Morris D.L., Rebelo L.R., Dieter P.A., Lee C. (2018). Validating intrinsic markers and optimizing spot sampling frequency to estimate fecal outputs. J. Dairy Sci..

[bib12] NASEM (National Academies of Sciences Engineering and Medicine) (2021).

[bib13] NRC (National Research Council) (2001).

[bib14] NRC (National Research Council) (2005).

[bib15] SAS Institute (2012). User's Guide: Statistics. Version 9.4.

[bib16] Siddons R.C., Paradine J., Beever D.E., Cornell P.R. (1985). Ytterbium acetate as a particulate-phase digesta-flow marker. Br. J. Nutr..

[bib17] Titgemeyer E.C. (1997). Design and interpretation of nutrient digestion studies. J. Anim. Sci..

[bib18] Van Soest P.J., Robertson J.B., Lewis B.A. (1991). Methods for dietary fiber, neutral detergent fiber, and nonstarch polysaccharides in relation to animal nutrition. J. Dairy Sci..

[bib19] Velásquez A.V., da Silva G.G., Sousa D.O., Oliveira C.A., Martins C.M.M.R., dos Santos P.P.M., Balieiro J.C.C., Rennó F.P., Fukushima R.S. (2018). Evaluating internal and external markers versus fecal sampling procedure interactions when estimating intake in dairy cows consuming a corn silage-based diet. J. Dairy Sci..

[bib20] Weiss W.P. (2017). A 100-Year Review: From ascorbic acid to zinc - Mineral and vitamin nutrition of dairy cows. J. Dairy Sci..

[bib21] Weiss W.P., Hansen S.L. (2024). Invited review: Limitations to current mineral requirement systems for cattle and potential improvements. J. Dairy Sci..

